# Supplementary material for: HIVprotI: an integrated web based platform for prediction and design of HIV proteins inhibitors
Source: J Cheminform. 2018 Mar 9;10:12. doi: 10.1186/s13321-018-0266-y (PMC5845081; doi:10.1186/s13321-018-0266-y)
Supplement: Supplementary file 2 — Additional file 2. Source code of HIVProtI web server. [file 13321_2018_266_MOESM2_ESM.zip › HIVprotI_Source-code/hivprotI/weka-3-6-6/doc/weka/core/converters/URLSourcedLoader.html]

URLSourcedLoader


---


|  |  |  |
| --- | --- | --- |
| **Overview** \| **Package** \| **Class** \| **Tree** \| **Deprecated** \| **Index** \| **Help** \| **Weka's home** | |  |
| **PREV CLASS**   **NEXT CLASS** | **FRAMES**    **NO FRAMES**     **All Classes** |  |
| SUMMARY: NESTED \| FIELD \| CONSTR \| METHOD | DETAIL: FIELD \| CONSTR \| METHOD |  |


---


## weka.core.converters Interface URLSourcedLoader

**All Known Implementing Classes:**: ArffLoader, LibSVMLoader, SVMLightLoader, XRFFLoader

---

``` public interface URLSourcedLoader ```

Interface to a loader that can load from a http url

**Version:**
:   $Revision 1.0 $

**Author:**
:   Mark Hall

---

| **Method Summary** | |
| --- | --- |
| `java.lang.String` | `retrieveURL()`             Return the current url |
| `void` | `setURL(java.lang.String url)`             Set the url to load from |

**Method Detail**

### setURL

```
void setURL(java.lang.String url)
            throws java.lang.Exception
```

:   Set the url to load from

    :   **Parameters:**: `url` - the url to load from **Throws:**: `java.lang.Exception` - if the url can't be set.

---


### retrieveURL

```
java.lang.String retrieveURL()
```

:   Return the current url

    :   **Returns:**: the current url


---


|  |  |  |
| --- | --- | --- |
| **Overview** \| **Package** \| **Class** \| **Tree** \| **Deprecated** \| **Index** \| **Help** \| **Weka's home** | |  |
| **PREV CLASS**   **NEXT CLASS** | **FRAMES**    **NO FRAMES**     **All Classes** |  |
| SUMMARY: NESTED \| FIELD \| CONSTR \| METHOD | DETAIL: FIELD \| CONSTR \| METHOD |  |


---
